# Antimony Nanoparticles Encapsulated in Self-Supported Organic Carbon with a Polymer Network for High-Performance Lithium-Ion Batteries Anode

**DOI:** 10.3390/nano12142322

**Published:** 2022-07-06

**Authors:** Zhaomin Wang, Fanming Zeng, Dongyu Zhang, Yabin Shen, Shaohua Wang, Yong Cheng, Chun Li, Limin Wang

**Affiliations:** 1 School of Materials Science and Engineering, Changchun University of Science and Technology, Changchun 130022, China; zmwang@ciac.ac.cn; 2 Collaborative Innovation Center of Optical Materials and Chemistry, Changchun University of Science and Technology, Changchun 130022, China; 3 State Key Laboratory of Rare Earth Resource Utilization, Changchun Institute of Applied Chemistry, CAS, Changchun 130022, China; dyzhang@ciac.ac.cn (D.Z.); ybshen@ciac.ac.cn (Y.S.); shwang@ciac.ac.cn (S.W.); cyong@ciac.ac.cn (Y.C.); 4 Key Laboratory of Preparation and Applications of Environmental Friendly Materials, Jilin Normal University, Ministry of Education, Changchun 130103, China; 5 State Key Laboratory of Metastable Materials Science and Technology, Yanshan University, Qinhuangdao 066004, China

**Keywords:** Sb/C, anode, alloying-conversion action, lithium-ion batteries

## Abstract

Antimony (Sb) demonstrates ascendant reactive activation with lithium ions thanks to its distinctive puckered layer structure. Compared with graphite, Sb can reach a considerable theoretical specific capacity of 660 mAh g^−1^ by constituting Li_3_Sb safer reaction potential. Hereupon, with a self-supported organic carbon as a three-dimensional polymer network structure, Sb/carbon (3DPNS-Sb/C) composites were produced through a hydrothermal reaction channel followed by a heat disposal operation. The unique structure shows uniformitarian Sb nanoparticles wrapped in a self-supported organic carbon, alleviating the volume extension of innermost Sb alloying, and conducive to the integrality of the construction. When used as anodes for lithium-ion batteries (LIBs), 3DPNS-Sb/C exhibits a high invertible specific capacity of 511.5 mAh g^−1^ at a current density of 0.5 A g^−1^ after 100 cycles and a remarkable rate property of 289.5 mAh g^−1^ at a current density of 10 A g^−1^. As anodes, LIBs demonstrate exceptional electrochemical performance.

## 1. Introduction

What accompanies the swift advancement of various intelligent mobile appliances is the enhancement of energy requirements, and LIBs have become attractive energy storage and conversion devices [1,2,3,4]. Finding electrodes with superior capacity is one of the most diffusely researched subjects in the domain of LIBs since the invertible ability is diametrically relevant to the useful life of the cell [5,6,7,8,9]. Sb-based anode material has received much attention and combines Li to form the Li_3_Sb alloy and gives rise to an excellent theoretical specific capacity (660 mAh g^−1^) [10,11,12,13,14,15,16]. Furthermore, Sb is a member of the most prospective anode materials for LIBs, which can be alloyed with Li at a low reaction potential of approximately 0.8 V [12]. Nevertheless, Li-ion insertion/extraction procedures lead to severe volume effect, which causes the prompt exacerbation of cycle property [11,17].

Therefore, numerous strategies have been utilized to mitigate these issues of Sb-based material anodes. For example, shrinking the grain diameter can curtail the Li-ion convey way and dramatically mitigate the mechanical stress during alloy reaction and thus moderate the pulverization trouble, which is a pervasive medium to improve the property of Sb-based anodes [11,18,19,20]. However, the machinery unsteadiness associated with the lithium alloy reaction cannot be entirely resolved only by reducing the particle dimension. The carbon matrix may undertake a rampart to adapt the polymerization and pulverization of active granules while enhancing the conductivity, which is deemed as a member of the prospective means to ameliorate the electrochemical property [14,21,22,23,24,25,26,27,28,29,30,31,32,33,34,35,36,37,38,39,40,41]. Noteworthily, since antimony is a heat-shrinking, cold-expanding metal, intermetallic systems (Sb-based alloys) can possess a powerful structural relationship with Li-ion, which leads to minor volume effects during the charge/discharge process [42,43,44,45,46]. Although the series of preparations mentioned above effectively buffer the volume effect and mechanical tension of Sb-based materials, the synthesis of nanoporous Sb-based composites through an uncomplicated and extensible method is still essential for practical application in LIBs.

Herein, an innovative 3DPNS-Sb/C nanoparticle anode material is fabricated based on the above discussions. It involves the uniformly in situ insertion of Sb nanoparticles into self-supported organic carbon, exploiting a manageable hydrothermal synthesis reaction and annealing treatment. The generation of the evenly distributed structure could be attributed to the facile reduction of sodium antimonate (NaSbO_3_) and the formidable binding interaction of the carbon network. The 3DPNS-Sb/C composites have the merits of the polymer network structure effect and high conductivity. In addition, the unique construction plays an appreciable role in enhancing the charge transfer kinetics and structure steadiness during the repeated insertion/deinsertion procedure of Li-ion, which ultimately exhibited excellent cyclability and rate property.

## 2. Experimental Section

### 2.1. Materials

Glucose (C_6_H_12_O_6_, CP, 99%), sodium antimonate (NaSbO_3_, 99.9%) and sodium polyacrylate ((C_3_H_3_NaO_2_)_n_, 99%) were stocked from Aladdin Reagent Co. Ltd. Shanghai, China. All the chemicals and solvents were exploited as acquired without further depuration. 

### 2.2. Synthesis of the3DPNS-Sb/C Composites

The 3DPNS-Sb/C composites were fabricated utilizing a common hydrothermal synthesis reaction and annealing treatment. Typically, 1 g NaSbO_3_ and 0.02 g (C_3_H_3_NaO_2_)_n_ were dispersed in a Teflon-lined autoclave with 100 mL aqueous liquor consisting of 3 g C_6_H_12_O_6_, and the autoclave was shut and conserved at 180 °C for 12 h, followed by a return to room temperature. Next, the as-synthesized precursor was subjected to an annealing process at 450 °C for 6 h with a warming speed of 3 °C min^−1^ under a perpetual high-purity Ar. After spontaneously dropping down to ambient temperature, the obtained product was denoted as 3DPNS-Sb/C-2. To assess the impact of the carbon content of the 3DPNS-Sb/C composites, two distinct C_6_H_12_O_6_ concentrations (m(C_6_H_12_O_6_) = 2.5/3.5 g) were also carried out while keeping other factors unchanged, which were denoted as 3DPNS-Sb/C-1 and 3DPNS-Sb/C-3, respectively.

### 2.3. Materials Characterization

X-ray diffraction (XRD, Bruker D8 Advance diffractometer using Cu Kα radiation (λ = 1.5418 Å)) was used to authenticate the component and crystal structure of the as-obtained 3DPNS-Sb/C composites. Field emission scanning electron microscopy (FESEM, Hitachi S-4800, Tokyo, Japan) and transmission electron microscopy (TEM, FEI Tecnai G2 S-Twin, Hillsboro, OR, America) were utilized to identify the morphology and structural characteristics. Thermogravimetric analysis (TGA) was executed utilizing a Q50 (Guangzhou, China) thermogravimetric analyzer from 25 through 800 °C at a velocity of 10 °C min^−1^ in an atmosphere of air. Nitrogen desorption/adsorption isotherms were assessed by nitrogen adsorption at 77 K using a Quadrachrome Adsorption Apparatus (Beijing, China). The Raman spectrum was acquired using a Renishaw Invia Raman microscope (Beijing, China). The X-ray photoelectron spectra (XPS) were recorded on a Thermo Scientific ESCALAB 250Xi (Shanghai, China) X-ray photoelectron spectrometer with a monochromatized Al-Kα X-ray (1486.6 eV) as the excitation source to estimate the apparent component.

### 2.4. Electrochemical Characterization

The anode electrode was prepared by mixing 80 wt.% 3DPNS-Sb/C, 10 wt.% acetylene black and 10 wt.% carboxymethyl cellulose sodium (CMC) with an appropriate amount of DI water as the solvent to produce a homogeneous phase of the slurry. The slurry was uniformly spread onto pure copper foil (of thickness 10 μm) current collector and dried at 60 °C for 6 h under vacuum conditions. Subsequently, the loaded collector was punched into a circular slice with an area of 1.13 cm^2^. The electrodes were then pressed using a stainless-steel metal disc to enhance the contact between the material and the Cu foil. The mass of material loading on each electrode was about 0.93–1.12 mg cm^−2^ (including the weight of acetylene black and the binder). The electrochemical assessments were executed using CR2025 coin-type batteries. Lithium foil was utilized as both the counter and the reference electrode, while the 3DPNS-Sb/C electrode was the working electrode. Polypropylene membrane (Celgard 2400) was employed as the separator for LIBs. The electrolyte was constituted of a solution of 1 M LiPF_6_ dispersed in a blend of dimethyl carbonate (DMC), diethyl carbonate (DEC) and ethylene carbonate (EC) (1:1:1 vol.%) with the addition of 10 vol.% fluoroethylene carbonate (FEC) for LIBs. The electrochemical properties of all the manufactured half-batteries was assessed by cyclic voltammetry (CV) measurement using the BioLogic VMP3 instrument. The charge/discharge performance was measured at room temperature with disparate current densities under the potential window of 0.01~2 V (vs. Li/Li^+^) employing the LAND CT2001A multichannel battery examination system. 

## 3. Results and Discussion

### 3.1. Experimental Synthesis Mechanism 

**Figure 1:**
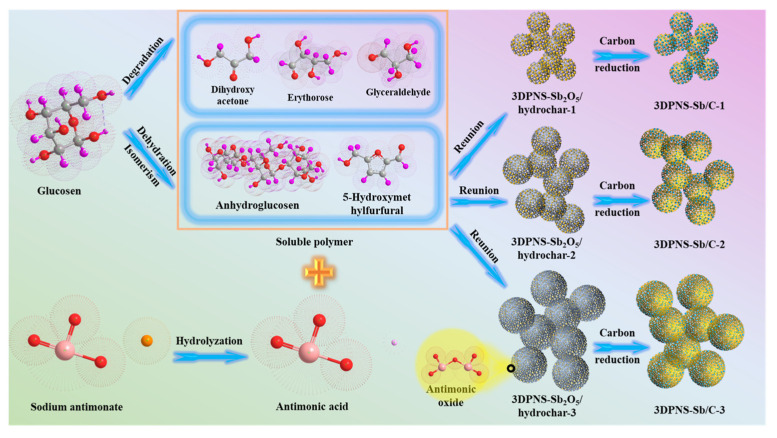
Schematic picture of the synthesis process of 3DPNS-Sb/C nanoparticle materials.

The prototypical design approach and synthesis path applied for manufacturing the 3DPNS-Sb/C nanoparticle materials are schematically demonstrated in Figure 1. In the first step, glucose molecules engender dihydroxyacetone, glyceraldehyde, erythrose, organic acids, aldehydes and other small molecular substances by cracking. On the other side, the glucose molecules produce anhydroglucose polymers via mutual dehydration or generate 5-hydroxymethylfurfural by self-isomerization. These molecules, of distinct sizes, are dehydrated under hydrothermal conditions and condense with each other to form the soluble polymer. [47] At the same time, the NaSbO_3_ is heated and hydrolyzed into antimonic acid (HSbO_3_) colloid, which is uniformly dispersed in the soluble glucose polymer under the action of (C_3_H_3_NaO_2_)_n_. With the temperature reaching a critical value, the soluble glucose polymer is progressively carbonized and the HSbO_3_ is gradually decomposed into antimonic oxide (Sb_2_O_5_) and ultimately, the 3DPNS-Sb_2_O_5_/hydrochar nanoparticle composites are obtained. [47] Subsequently, the Sb nanoparticles were evenly distributed in an organic carbon skeleton during the annealing treatment, which originated from the in-situ reduction reaction of Sb_2_O_5_ and hydrochar. 

### 3.2. Morphology Analysis 

**Figure 2:**
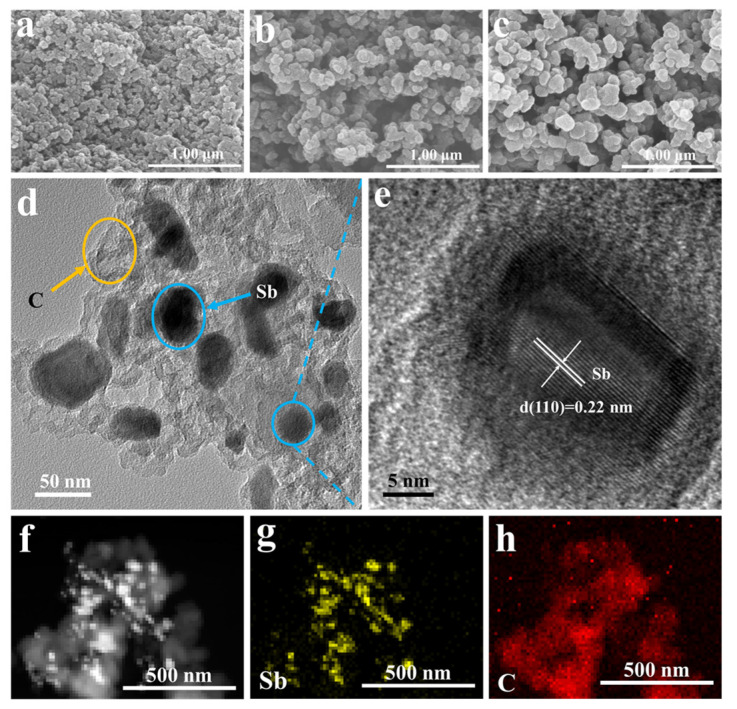
(**a**–**c**) SEM images of the 3DPNS-Sb/C-1, 3DPNS-Sb/C-2 and 3DPNS-Sb/C-3 composites, (**d**,**e**) TEM and HRTEM images of the 3DPNS-Sb/C-2 composites, (**f**) STEM image, (**g**) Sb and (**h**) C element mappings of the 3DPNS-Sb/C-2 composites.

**Figure 3:**
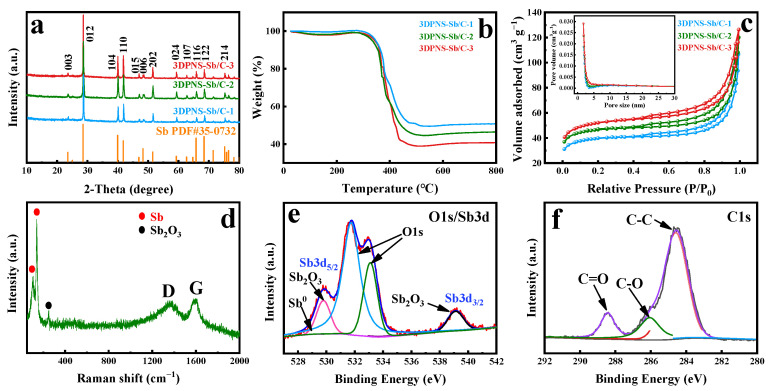
(**a**) XRD patterns of 3DPNS-Sb/C-1, 3DPNS-Sb/C-2 and 3DPNS-Sb/C-3. (**b**) TG curves of 3DPNS-Sb/C-1, 3DPNS-Sb/C-2 and 3DPNS-Sb/C-3. (**c**) Nitrogen adsorption–desorption isotherms and related pore dimension distribution curves of 3DPNS-Sb/C-1, 3DPNS-Sb/C-2 and 3DPNS-Sb/C-3. (**d**) Raman spectra of 3DPNS-Sb/C-2. XPS spectra of 3DPNS-Sb/C-2: (**e**) Sb 3d and O 1s, (**f**) C 1s.

The morphologies of the acquired 3DPNS-Sb/C composites are shown in Figure 2. As illustrated in Figure 2a–c, the 3DPNS-Sb/C composites clearly show an interconnected 3D polymer network framework structure and individual Sb/C nanoparticles with a diameter of about 50–200 nm. Furthermore, the size of this individual Sb/C nanoparticle enlarges with increasing carbon content. As shown in Figure 2d,e, the TEM and high-resolution TEM (HRTEM) figures (3DPNS-Sb/C-2) indicate the lattice fringes with an interval of 0.22 nm, coinciding with the (110) planes of hexagonal Sb, further verifying the high crystallinity of the Sb. In addition, the tiny Sb nanoparticles are equally distributed in a thin carbon layer structure, which can supply a more favorable appearance and curtail the diffusion interval for ions to inner pores. As revealed in Figure 2f–h, the detected element mappings, such as Sb and C, display a uniform dispersion in the 3DPNS-Sb/C-2 composites.

### 3.3. Microstructure and Component Analysis

The structure of 3DPNS-Sb/C composites is demonstrated by the XRD examination. As revealed in Figure 3a, the significant characteristic peak of 23.6°, 28.6°, 40°, 41.9°, 47°, 48.4°, 51.5°, 59.3°, 62.7°, 65.9°, 68.5° and 75.3° is admirably indexed to the (003), (012), (104), (110), (015), (006), (202), (024), (107), (116), (122) and (214) crystal face of elemental Sb (JCPDS 35-0732), respectively, which is consistent with the scrutinization in the HRTEM image. In addition, the quantitative component of the 3DPNS-Sb/C specimens is confirmed by TGA. In accordance with the TGA consequences in Figure 3b, the Sb content is computed to be 40.23, 36.79 and 32.51 wt.% for 3DPNS-Sb/C-1, 3DPNS-Sb/C-2 and 3DPNS-Sb/C-3 composites, respectively. The subsequent weight increase corresponds to Sb oxidation [48,49]. The specific surface acreage and the pore size distribution of 3DPNS-Sb/C samples are afterwards explained via nitrogen adsorption/desorption isotherm measuring. Figure 3c demonstrates that the isotherms present type IV features, which means they belong to mesoporous substances [48]. The BET-specific surface acreage of 3DPNS-Sb/C-1, 3DPNS-Sb/C-2 and 3DPNS-Sb/C-3 composites are 136.7, 141.5 and 147.8 m^2^ g^−1^, respectively, which could be owing to the coordination of the large surface area of the 3D polymer network structure and small Sb nanoparticles. The relevant pore diameter distribution curve (inset in Figure 3c) also evidences a mesoporous constitution, and the pore dimension ratio (3.5–4.8 nm) is evidenced. For the cell electrode, the powerful specific surface area and mesoporous construction of 3DPNS-Sb/C samples can accelerate the pervasion of Li-ions and electrons. The Raman spectroscopy analysis was conducted further to inspect the structural characteristics of the 3DPNS-Sb/C-2 composites, with the acquired spectrogram revealed in Figure 3d. Two typical bands located at 107 and 139 cm^−1^ are associated with the Sb phase in the nanohybrid [26,50,51,52,53]. Moreover, Raman scattering measurement analysis confirmed that the Sb nanoparticles contain traces of Sb_2_O_3_ [54]. The peak at 1346 cm^−1^ indicates the disarray-induced D-band, which connects with flaws in the sp^2^ lattice construction of carbon. The peak at 1592 cm^−1^ correlates with the graphitic G-band, which insinuates the sp^2^ lattice of carbon. The above results indicate the amorphous character of the carbon contained in the 3DPNS-Sb/C-2 composites [23,49]. The surface chemical composition of the 3DPNS-Sb/C-2 composites was also studied by XPS characterization, and the Sb and C spectrums of the 3DPNS-Sb/C-2 composites are demonstrated in Figure 3e,f, which absolutely correspond to the EDX mapping aforementioned. Among these, two peaks at 531.7 eV and 533.1 eV are from the O 1s core-level XPS spectrum, while the other peaks arise from the XPS spectrum of Sb 3d. In particular, the two peaks centered at 539.23 eV (Sb 3d_3/2_) and 529.85 eV (Sb 3d_5/2_) represent Sb_2_O_3_. Meanwhile, the peak appearing at 528.7 eV (Sb 3d_5/2_) represents metallic Sb (Figure 3e) [23,28,33,55]. Hence, this result confirms the partial oxidation of Sb by oxygen. Meanwhile, the high-resolution C 1s spectrum displayed in Figure 3f could be fitted into three peaks correlated with C-C (284.55 eV), C-O (286.01 eV) and C=O (288.44 eV) bonds [55,56,57].

## 4. Electrochemical Evaluation in LIBs

**Figure 4:**
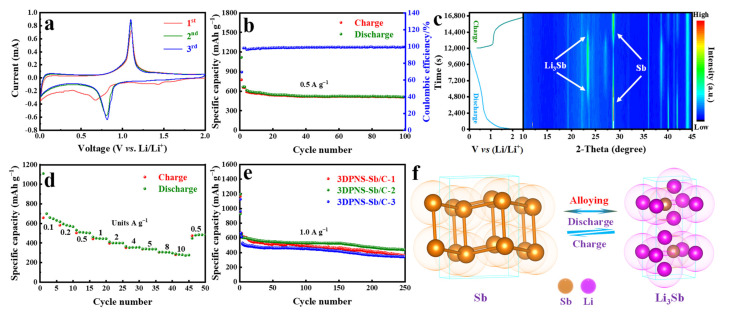
(**a**) CV curves of 3DPNS-Sb/C-2 electrodes at a scan rate of 0.1 mV s^−1^, (**b**) Cycle performance of 3DPNS-Sb/C-2 electrodes at 0.5 A g^−1^ for 100 cycles, (**c**) In-situ XRD of 3DPNS-Sb/C-2 electrodes, (**d**) Rate capability of 3DPNS-Sb/C-2 electrodes at current densities from 0.1 to 10 A g^−1^, (**e**) Cycle performance of 3DPNS-Sb/C-1, 3DPNS-Sb/C-2 and 3DPNS-Sb/C-3 electrodes at 1 A g^−1^ for 250 cycles, (**f**) The crystal structures of the active Sb in 3DPNS-Sb/C in the charge/discharge course.

The cyclic voltammetry (CV) curves of the 3DPNS-Sb/C-2 electrode for the initial three cycles are demonstrated in Figure 4a. In the primary cathode scanning, the capacious summit between 0.8 and 0.6 V could be ascribed to a suite of Li-insertion reactions, containing the reaction of metallic Sb to alloyed Li_3_Sb and the generation of an SEI film on the cover of the electrode from electrolyte decomposition. During the initial invertible anode scanning, the anode summit at around 1.14 V conforms to the Li-extraction reaction of Li_3_Sb to metallic Sb [11,17,58]. In following cyclings, peaks tend to overlap, suggesting excellent electrochemical invertibility of the 3DPNS-Sb/C-2 samples in the lithiation–delithiation procedure. Subsequently, it still displays an illustrious invertible specific capacity of 511.5 mAh g^−1^ at a current density of 0.5 A g^−1^ after 100 cycles, with a primary charge/discharge specific capacity of 775.3/1117.8 mAh g^−1^ and a first coulombic efficiency (CE) of 69.35% (Figure 4b). Figure S1 shows the SEM images of 3DPNS-Sb/C-2 after 100 repeated cycles at a current density of 0.5 A g^−1^. Distinctly, the structure is nearly maintained, suggesting excellent structural stability. Moreover, with the unique network constructure of 3DPNS-Sb/C-2 composites, a pronouncedly high-rate capacity is acquired (Figure 4d). Though the current density enhances from 0.1 to 10 A g^−1^, it could release a high specific capacity of 289.5 mAh g^−1^. Noteworthily, the gaps between charge/discharge of the 3DPNS-Sb/C-2 composites reduce slightly with the current density increase, which signifies the weakness of polarization and mechanical effect in the cycling procedure [59,60]. Figure 4e displays the cycle property of the 3DPNS-Sb/C-1, 3DPNS-Sb/C-2 and 3DPNS-Sb/C-3 electrodes, and the reversible specific capacities are still 359.3, 440.5 and 341 mAh g^−1^ at a current density of 1 A g^−1^ after 250 cycles, respectively. Obviously, the 3DPNS-Sb/C-2 electrode exhibits superior cycle stability and reversible specific capacity. Furthermore, 3DPNS-Sb/C also reveals surpassing performance in comparison with the commercial LIBs anode materials (graphite, LTO) (Figure S2). In contrast with other statements on diverse Sb-C positive materials, the 3DPNS-Sb/C composites also show excellent electrochemical properties (Table 1). 

To further research the phase changes and the reaction mechanism, the 3DPNS-Sb/C-2 electrodes at diverse conditions of charge/discharge during the cycle were inspected using in-situ XRD. As shown in Figure 4c, the phase change of Sb could be watched from the peak intention variation on the contour map of the in-situ XRD consequence. The primary phase is at 28.7°, matched with the (012) crystal plane of Sb (JCPDS: 35-0732). As the discharge procedure continues, the diffraction peaks of Sb crystal progressively step down with alloying reaction between Li^+^ and Sb to firstly form Li_x_Sb (x ≤ 3) phase located at 23.4°. In the reversible charge process, the diffraction peaks of the Li_3_Sb phase gradually disappear with the representation of the Sb phase, manifesting the arising of a dealloying reaction. Especially, Figure 4f interprets the alloying mechanism of the active Sb in the 3DPNS-Sb/C composites, which could be deemed as a type of alloying-typed material with a better electrochemical property.

## 5. Conclusions

In conclusion, the 3DPNS-Sb/C composites are fabricated with Sb nanoparticles uniformly embedded in the 3D polymer network structure via an uncomplicated and controllable synthetic medium. Based on the 3D polymer network structure, the 3DPNS-Sb/C composites employed as an anode display excellent electrochemical properties in LIBs. Specifically, they demonstrate a high invertible specific capacity of 511.5 mAh g^−1^ at a current density of 0.5 A g^−1^ after 100 cycles and a remarkable rate property of 289.5 mAh g^−1^ at a current density of 10 A g^−1^. This study explicitly demonstrates the promising potential of the 3DPNS-Sb/C composites as well-performaning LIBs anode.

## Figures and Tables

**Table 1:** Contrast of the electrochemical properties of 3DPNS-Sb/C composites (this work) and various reported Sb-C as anodes for LIBs.

Material	Reversible Capacity/mAh g^−1^	Current Density (mA g^−1^)	Areal Mass Loading (mg cm^−2^)	Batteries	Ref.
Hollow Sb Nanoparticles	615/100th cycles	120		Li-ion	[61]
Sb nanoparticles	120/70th cycles	120		Li-ion	[62]
Sb-carbon nanocomposite	550/250th cycles	230	1.07–1.11	Li-ion	[51]
Sb/C composite fibers	315.9/100th cycles	100		Li-ion	[63]
Sb HNSs	627.3/50th cycles	100		Li-ion	[12]
Sb nanocrystals	600/100th cycles	660		Li-ion	[11]
Spherical Sb/C Composites	590/80th cycles	100	1	Li-ion	[49]
Sb@C nanosponges	447.1/500th cycles	660	1.5	Li-ion	[23]
Sb/C micro-/nanohybrid	793/100th cycles	66		Li-ion	[26]
Sb@C composites	598.6/100th cycles	100	1.132	Li-ion	[21]
Sb/C/G nanocomposites	413/700th cycles	1000	1.0	Li-ion	[32]
Sb/NPC	556/100th cycles	200	1.00	Li-ion	[14]
Sb@C composites	280/500th cycles	100	1.35	Li-ion	[30]
Sb@CNFs	394.5/2000th cycles	2000	0.8	Li-ion	[28]
Sb2Se3/Sb/C nanofibers	764/300th cycles	100		Li-ion	[33]
Sb@C/EG	486/600th cycles	1000	0.5	Li-ion	[34]
Ni-Co-Sb/C Nanosphere	354/100th cycles	100	~0.55	Li-ion	[31]
Sb@C	525/400th cycles	500	1.2–1.5	Li-ion	[24]
3DPNS-Sb/C composites	511.5/100th cycles	500	0.93–1.12	Li-ion	this work (586 Wh L^−1^) (ICE: 69.35%)

## Data Availability

Not applicable.

## Supplementary Materials

The following supporting information can be downloaded at: https://www.mdpi.com/article/10.3390/nano12142322/s1, Figure S1: SEM images of 3DPNS-Sb/C-2 after 100 cycles.; Figure S2: (a) Cycle performances of Li_4_Ti_5_O_12_ at 0.5 A g^−1^ for 50 cycles, (b) Cycle performances of graphite at 0.5 A g^−1^ for 50 cycles.
