# Supplementary material for: Antimony Nanoparticles Encapsulated in Self-Supported Organic Carbon with a Polymer Network for High-Performance Lithium-Ion Batteries Anode
Source: Nanomaterials (Basel). 2022 Jul 6;12(14):2322. doi: 10.3390/nano12142322 (PMC9316927; doi:10.3390/nano12142322)
Supplement: Supplementary file 1 [file nanomaterials-12-02322-s001.zip › nanomaterials-1763190-supplementary.pdf]

Supplementary Materials

Antimony Nanoparticles Encapsulated in Self-Supported Organic Carbon with a Polymer Network for High-Performance Lithium-Ion Batteries Anode

Zhaomin Wang [1,2], Fanming Zeng [1,2,*], Dongyu Zhang [3], Yabin Shen [3], Shaohua Wang [3], Yong Cheng [3,4,5], Chun Li [1,2,*] and Limin Wang [3,*]

[1] School of Materials Science and Engineering, Changchun University of Science and Technology, Changchun 130022, China; zmwang@ciac.ac.cn
[2] Collaborative Innovation Center of Optical Materials and Chemistry, Changchun University of Science and Technology, Changchun 130022, China
[3] State Key Laboratory of Rare Earth Resource Utilization, Changchun Institute of Applied Chemistry, CAS, Changchun 130022, China; dyzhang@ciac.ac.cn (D.Z.); ybshen@ciac.ac.cn (Y.S.); shwang@ciac.ac.cn (S.W.); cyong@ciac.ac.cn (Y.C.)
[4] Key Laboratory of Preparation and Applications of Environmental Friendly Materials, Jilin Normal University, Ministry of Education, Changchun 130103, China
[5] State Key Laboratory of Metastable Materials Science and Technology, Yanshan University, Qinhuangdao 066004, China
* Correspondence: zengfm@126.com (F.Z.); lichun1210@163.com (C.L.); lmwang@ciac.ac.cn (L.W.)

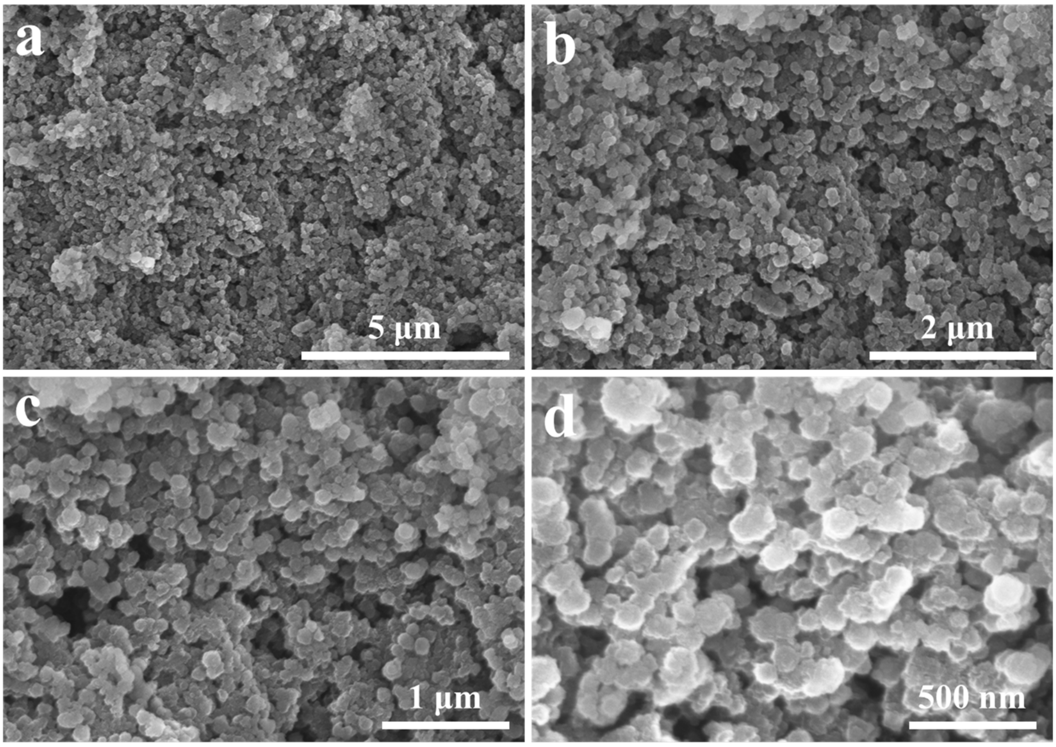

Figure S1. (**a**–**d**) SEM images of 3DPNS-Sb/C-2 after 100 cycles.

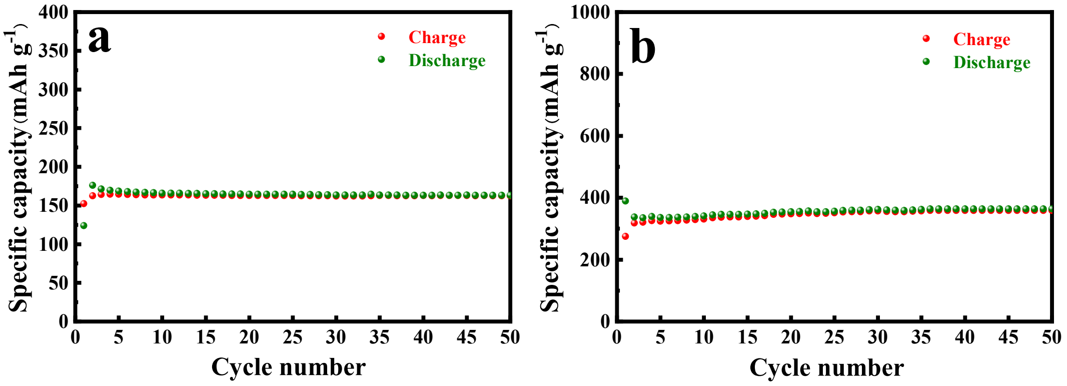

Figure S2. (**a**) Cycle performances of $Li_4Ti_5O_{12}$ at 0.5 A g^{-1} for 50 cycles, (**b**) Cycle performances of graphite at 0.5 A g^{-1} for 50 cycles.
